# Perceived Stress and Indicators of Burnout in Teachers at Portuguese Higher Education Institutions (HEI)

**DOI:** 10.3390/ijerph17093248

**Published:** 2020-05-07

**Authors:** Renata Teles, Antonio Valle, Susana Rodríguez, Isabel Piñeiro, Bibiana Regueiro

**Affiliations:** 1 Clinical Psychologist, 3700-025 São João da Madeira, Portugal; renatteles@gmail.com; 2 Psychology Departament, University of A Coruña, 15001 A Coruña, Spain; antonio.valle@udc.es (A.V.); susana@udc.es (S.R.); ipineiro@udc.es (I.P.); 3 Pedagogy and Didactics Departament, University of Santiago de Compostela, 15705 Santiago de Compostela, Spain

**Keywords:** perceived stress, burnout syndrome, emotional exhaustion, depersonalization, personal accomplishment, higher education professor

## Abstract

The aim of this study was to examine the phenomena of burnout and perceived stress in teachers at Higher Education Institutions, as this professional class is one of the most affected by high levels of stress. A sample of 520 university teachers was used, of which 339 (65.2%) were women. The Maslach Burnout Inventory (MBI) was used to measure burnout, and the Perceived Stress Scale (PSS) was used to measure perceived stress. A sociodemographic data questionnaire produced by the authors was also applied, which consisted of questions about age, sex, experience in the teaching profession and the participants’ teaching areas. The results indicated that university teachers over 60 years old exhibited lower levels of perceived stress, as did teachers with more teaching experience (30 years or more), and those with less experience (less than 10 years). Women exhibited higher levels of perceived stress than men. Women also scored higher levels of Emotional Exhaustion in the burnout dimensions, whereas teachers will less experience (under 10 years) and teachers with more experience (more than 30 years) had the lowest scores in this dimension. Through an examination of the relation between perceived stress and the burnout dimensions, we concluded that perceived stress was directly proportional to emotional exhaustion and depersonalization; and was inversely proportional to personal accomplishment. A total of 31.3% of the variance in burnout was explained by perceived stress.

## 1. Introduction

Nowadays, university teachers must interact constantly with students, they must maintain high levels of professional performance, and they have to meet goals and comply with deadlines at the same time as delivering regular curricular obligations and fulfilling other demands of the job. These are all factors that increase the stress of the professionals in this group. Teaching at the higher education level is usually described as involving a certain degree of complexity since it takes place along a triple axis, involving teaching–research–extending and affects political, social, intellectual, psychological and pedagogical viewpoints [1]. 

In recent years, research on burnout has taken a particular interest in the academic field [2]. Several researchers [3,4,5,6] agree that teaching is one of the professions most affected by burnout Syndrome, with many cases presenting critical levels of stress. For this reason, it is considered a high-risk professional class [6]; teachers are the most commonly studied group in the burnout literature, given that they present record levels of exhaustion and fatigue [7,8]. 

Pinto, Lima and Lópes [9] reported worrying data about professional malaise, pointing out that 54% of the respondents considered the activity of teachers to be very or extremely stressful, 6.3% showed high levels of burnout and 30.4% were at risk of progressing to a “Pre-Burnout” condition. 

Different authors have approached Burnout Syndrome (BS) using different models [3] such as the Maslach model [10]. In this study, we chose the Maslach model, which characterizes the syndrome in three dimensions which are conceptually distinct but empirically related: Emotional Exhaustion (EE), Depersonalization (DP) and a low sense of Personal Accomplishment (PA) [10]. 

Emotional Exhaustion arises as emotional resources are depleted; teachers feel they are no longer able to give of themselves at a psychological level. Depersonalization occurs when teachers develop negative cynical attitudes and feelings about their students. Reduced Personal Accomplishment refers to the tendency for teachers to evaluate themselves negatively, particularly in regard to their work with students [11]. 

Therefore, the well-being of higher education teachers is one of the key aspects of the success of higher education institutions and, consequently, of the success of students. This fact is the reason underlying the relevance of analyzing the prevalence of perceived stress and burnout in teachers, as well as learning about the problems of some risk factors directly associated with it. Exhaustion is extremely disabling and distressing in the life of every teacher, reducing their ability to work, develop expertise, and share their knowledge. However, not enough is known about this problem. 

### 1.1. Perceived Stress Related to Burnout Dimensions

The relationships between perceived stress and the dimensions of burnout have been investigated in many studies [12]. Several have demonstrated significant relationships between perceived stress and the three subscales. 

Smith’s proposed theoretical framework has received substantial support and has been used as the basis for much of the contemporary research on burnout. Smith’s model asserts that personal and situational characteristics influence the perception of stress, and perception of stress, in turn, influences the level of burnout [12].

To better illustrate what research has demonstrated in this area, attention must be given to the study by a group of Mexican researchers who concluded that, in university teachers, perceived stress was significantly correlated with the dimensions of Burnout Syndrome [13].

Mota Cardoso et al. [4] surveyed 2108 Portuguese university teachers from all over the country to assess their stress levels. The results of the study showed that 34.8% of the respondents presented signs of emotional exhaustion, 84.2% had signs and symptoms of a lack of professional accomplishment, and 6.3% exhibited depersonalization. The study also showed that 6.3% to 34.8% of the participants could have signs and symptoms characteristic of Burnout Syndrome, exhibiting moderate to severe burnout.

Hendrix, Acevedo and Hebert [14] found perceived stress to be a significant predictor of emotional exhaustion. Emotional exhaustion has consistently been reported as a primary factor in burnout because of feelings of despair, isolation, exhaustion, and being overwhelmed. 

Due to current policies, university teachers have been increasingly required to take on more tasks, functions and activities in addition to their regular teaching obligations. Despite teachers reporting lower levels of emotional exhaustion when some of their time is spent performing administrative duties than when working in direct contact with students [14], these factors, associated with personal factors, increase levels of Emotional Exhaustion. This can trigger a prolonged stress response, or acute stress, and the possible development of emotional exhaustion, which if it persists, can lead to Burnout Syndrome.

The remaining burnout dimensions, Personal Accomplishment and Depersonalization, have also been shown to demonstrate significant relationships to perceived stress [14].

From this perspective, everything points to perceived stress as one of the most important variables in burnout. 

### 1.2. Moderating Variables of Burnout Dimensions

As noted by Vallejo-Martín, Aja and Plaza [15], there are various moderating variables that can affect the experience of burnout and the perceived stress. These include sex and age, which have been assessed in previous research, but without a clear effect and with contradictory results.

In regards to sex, various studies have demonstrated its clear influence in perceived stress. Most research has indicated higher levels of perceived stress in women than in men [16,17,18]. When it comes to burnout dimensions, it is not so clear. In one study with Turkish pre-school teachers, which also used the Maslach Burnout Inventory (MBI), it was reported that women presented higher levels in Depersonalization and Emotional Exhaustion [19]. However, other studies have not produced the same findings. For example, Maslach and Jackson [10] and Marenco-Escuderos et al. [20] stated that men were more likely to develop Depersonalization. Previous research has also shown that men tend to report slightly higher scores on depersonalization than women [21].

Hendrix, Acevedo, and Hebert [14] found that Emotional Exhaustion was reported to be higher in women and in older individuals. Age seems to be a significant variable to study. Guedes and Gaspar [22] noted that teachers aged 50 or over were more frequently affected by burnout than teachers who were under 30. Furthermore, higher levels of Emotional Exhaustion and Depersonalization were observed in older professionals. 

In summary, because the professional class of university teachers has a high propensity to develop signs and symptoms of stress, we feel the need for this study to improve our understanding of burnout in the Portuguese context. The development of research, such as the one presented here, can contribute to providing a view of the map of occupational health and safety at work for Portuguese higher education teachers. This knowledge will provide the basis for designing prevention and intervention proposals with the teachers who are most at risk of developing burnout to avoid the strong impact it can have on the teaching activity and the students’ learning processes.

The study has an overall objective and two specific goals. The general objective is to understand the phenomena of burnout and perceived stress in Portuguese higher education teachers. The first specific goal is to examine the differences in perceived stress according to the sociodemographic variables included in the study (sex, age, teaching experience, and knowledge area) to look at the extent of the relationships between these variables and perceived stress. The second specific goal is to examine the relationship between perceived stress and the three dimensions of burnout: Emotional Exhaustion, Depersonalization, and Personal Accomplishment. 

We have formulated the following hypotheses:

**H1:**  *We expect to find statistically significant differences in perceived stress according to the sociodemographic variables, such as sex, age, and teaching experience*.

**H2:**  *We expect to find statistically significant relations between perceived stress and the three burnout subscales*.

## 2. Materials and Methods 

### 2.1. Participants and Procedure

The sample was drawn from the population of the teachers from Higher Education Institutions in Portugal (*n* = 32,346). The sampling followed a simple random method, based on an exhaustive and up-to-date database of all HEIs in Portugal and their teaching staff at the time of the study. A total of 524 questionnaires were received. Of those, 4 were immediately eliminated for being incomplete or containing incorrect information. The final sample comprised 520 respondents. Table 1 shows the distribution of the participants in terms of various significant variables.

The survey and the two scales were emailed to all HEI teachers in Portugal, with a request that they be completed and returned. It took respondents approximately 30 min to complete the application. Participants were informed that there were no right or wrong answers, that their personal data was confidential and would not be used in research or publications, which would only include quantitative and statistical data. Participants were also asked to answer all questions honestly, as the aim of the research was to understand the general state of burnout in higher education teachers in Portugal. The study was conducted according to the guidelines of the Ethics Committee at the University of A Coruña, Spain (https://www.udc.es/en/investigacion/etica/), with written informed consent from all participants, as established in the Helsinki Declaration.

### 2.2. Instruments

The instruments used in the study were the Maslach Burnout Inventory [23] and the perceived stress Scale [9]. Authorization was sought for the use of both questionnaires. A third instrument was added which was a sociodemographic questionnaire, prepared by the authors, consisting of questions about age (“under 40”, “40 to 49”, “50 to 59”, “over 60”), sex (women, men), years of experience in the teaching profession (“less than 10 years”, “10 to 19 years”, “20 to 29 years”, and “30 years or more”), and the knowledge areas taught (“Social Sciences”, “Health Sciences”, “Technological Sciences”, “Arts and Humanities” and “Others”).

The Perceived Stress Scale (PSS) used was the version translated and adapted by Mota-Cardoso, Araújo, Ramos, Gonçalves, and Ramos [4]. It has 14 items with responses given on a Likert scale with 5 alternatives, ranging from 0 to 4 points; 0 = “never”, 1 = “almost never”, 2 = “sometimes”, 3 = “very often” and 4 = “many times” (with Cronbach’s alpha α = 0.91) (for example: “In the last month, how often have you felt nervous or stressed?”, “In the last month, how often have I had to deal with small irritating events?”). 

The Maslach Burnout Inventory (MBI) used was also the version translated and adapted by the Institute for Stress Prevention and Occupational Health (IPSSO) [4]. This questionnaire examines the three dimensions that address the sociopsychological characteristics of “Burnout Syndrome”, namely: emotional exhaustion (EE—9 items; example, “I feel emotionally exhausted by my work”), personal accomplishment (PA—8 items; example. “I feel that I deal with my students’ problems very effectively”) and depersonalization (DP—5 items; example, “I feel that students blame me for some of their problems”).

Responses to the items are on a 7-point Likert scale, with 0 = “never”, 1 = “a few times a year or less”, 2 = “once a month or less”, 3 = “a few times a month”, 4 = “once a week”, 5 = “a few times a week”, and 6 = “every day” (Cronbach’s Alpha for Emotional Exhaustion α = 0.92, for Personal Accomplishment α = 0.81, indicators of internal consistency and for Depersonalization α = 0.71, which indicates substantial internal consistency).

### 2.3. Data Analysis

The data were analyzed in two stages. Firstly, using Analysis of Variance (ANOVA), we examined the differences in perceived stress based on the sociodemographic variables included in the study (sex, age, teaching experience, knowledge area taught) to understand the extent of the relationships between these variables and perceived stress.

Secondly, using Multivariate Analysis of Covariance (MANCOVA), we examined the relationship between perceived stress (independent variable) and the three burnout dimensions: Emotional Exhaustion, Depersonalization, and Personal Accomplishment (dependent variables). Those variables which demonstrated statistically significant relationships with perceived stress in the prior analysis of variance were included in the model in order to control for their effects. The independent variable (perceived stress) has three levels: Low Perceived Stress: scores below the 33 rd percentile; Average Perceived Stress: scores between the 33rd and 66th percentiles; and High Perceived Stress: scores above the 66th percentile).

The partial eta-squared coefficient (η_p_^2^) was used as a measure of effect size, as it is one of the most commonly used procedures in education research [24]. The criteria set in the classic work by Cohen (1988) were used to interpret the effect sizes. According to those criteria, an effect is small when η_p_^2^ = 0.01 (*d* = 0.20), moderate when η_p_^2^ = 0.059 (*d* = 0.50), and the effect size is large when η_p_^2^ = 0.138 (*d* = 0.80).

## 3. Results 

### 3.1. Differences in Perceived Stress Based on Various Sociodemographic Variables and Teachers’ Professional Situations

In terms of sociodemographic variables, the results indicated statistically significant differences in perceived stress depending on a teacher’s age (F_(3516)_ = 2.49, *p* < 0.05; *η_p_^2^* = 0.014), although the effect size was small. As Table 2 shows, the levels of perceived stress were relatively constant in teachers up to 49 years of age, and declined beyond that. Specifically, those over 60 reported lower levels of perceived stress.

There were statistically significant differences found in perceived stress according to sex (*F*_(1518)_ = 8.71, *p* < 0.01; *η_p_^2^* = 0.017). Female teachers reported significantly higher levels of perceived stress compared to male teachers, although the effect size was small. The results also indicated statistically significant differences in perceived stress depending on the amount of teaching experience (*F*_(3516)_ = 2.90, *p* < 0.05; *η_p_^2^* = 0.017). Teachers with less than 10 years of experience and those with at least 30 years of experience reported the lowest levels of perceived stress. Teachers with between 10 and 29 years of experience reported slightly higher levels. Finally, the results indicated that there were no statistically significant differences in perceived stress according to the knowledge areas taught (*F*(_4515)_ = 0.07, *p* = 0.990; *η_p_^2^* = 0.001), with the mean scores in the different areas being very similar.

### 3.2. Differences in Burnout in Relation to the Different Levels of Perceived Stress (Controlling the Effect of Covariates: Sex, Age, Years of Service) 

At the multivariate level, controlling for the effect of sex, age, and experience, the results demonstrated statistically significant differences in the burnout dimensions taken as a whole depending on perceived stress (λ_Wilks_ = 0.472, *F* (61022) = 77.50; *p* < 0.001, η_p_^2^ = 0.313). According to the criteria set out above, the effect size was large.

Sex was found to be significantly related to the three dimensions that make up burnout when taken together (λ_Wilks_ = 0.970, *F* (3512) = 5.27; *p* < 0.001, η_p_^2^ = 0.030), albeit with a small effect size. There were statistically significant differences by sex in Emotional Exhaustion (*F*_(1518)_ = 5.94, *p* < 0.05; *η_p_^2^* = 0.011), but there were no sex differences in Depersonalization (*F*_(1518)_ = 3.29, *p* = 0.07; *η_p_^2^* = 0.006) or in Personal Accomplishment (*F*_(1518)_ = 0.68, *p* = 0.41; *η_p_^2^* = 0.001). In other words, women seemed to present significantly higher levels of Emotional Exhaustion than men (see Table 2).

Teaching experience was also found to be significantly related to the three burnout dimensions when taken together (λ_Wilks_ = 0.975, *F* (3512) = 4.37; *p* < 0.01, η_p_^2^ = 0.025), also with a small effect size. There were statistically significant differences in Emotional Exhaustion (*F*_(3516)_ = 7.78, *p* < 0.001; *η_p_^2^*= 0.043) depending on years of experience. However, there were no significant differences according to experience in Depersonalization (*F*_(3516)_ = 0.20, *p* = 0.893; *η_p_^2^* = 0.001) or in Personal Accomplishment (*F*_(3516)_ = 1.22, *p* = 0.302; *η_p_^2^*= 0.007). The lowest scores in Emotional Exhaustion were reported from teachers with less than 10 years or more than 30 years of experience of teaching (see Table 3).

The age covariable was not found to be significantly related to the three dimensions making up burnout when taken together (λ_Wilks_ = 0.990, *F* (3512) = 1.73; *p* = 0.160, η_p_^2^ = 0.010). 

#### 3.2.1. Perceived Stress and Emotional Exhaustion 

The results demonstrated statistically significant differences in Emotional Exhaustion according to the various levels of perceived stress in the respondents (*F*_(2517)_ = 244.17, *p* < 0.001; *η_p_^2^* = 0.486), with a large effect size. As Table 3 shows, Emotional Exhaustion increased progressively as the levels of perceived stress increased. In addition, the increase in Emotional Exhaustion was much more marked when the levels of perceived stress were high. 

#### 3.2.2. Perceived Stress and Depersonalization 

The results indicated that there were statistically significant differences in Depersonalization depending on the various levels of perceived stress (*F*_(2517_) = 55.31, *p* < 0.001; *η_p_^2^* = 0.176), with an effect size that was large but somewhat smaller than for Emotional Exhaustion. As Table 3 shows, the degree of Depersonalization increased progressively as perceived stress levels increased. However, the results in Depersonalization were much lower than the mean scores for Emotional Exhaustion.

#### 3.2.3. Perceived Stress and Personal Accomplishment

The results also indicated statistically significant differences in Personal Accomplishment depending on the various levels of perceived stress (*F*_(2517)_ = 36.64, *p* < 0.001; *η_p_^2^* = 0.133), also with a large effect size. As Table 3 shows, Personal Accomplishment decreased progressively as the levels of perceived stress increased. In this case, the highest degree of Personal Accomplishment was in teachers who reported the lowest perceived stress. Conversely, the lowest levels of Personal Accomplishment were in teachers reporting the highest levels of perceived stress.

## 4. Discussion

Teaching is one of the professions that is most often associated with high levels of stress due to the demands of the current teaching role and in the face of constant change and the need for innovation [4]. Teachers are different from other occupational categories because of their teaching-specific demands and resources [25,26], hence the importance of this study. 

With respect to perceived stress, the results showed that female teachers exhibited higher levels of perceived stress than male teachers. We also found that university teachers who are over 60 reported lower levels of perceived stress. Another notable result was that teachers with the most experience (30 years or more) and teachers with the least experience (less than 10 years) reported the lowest levels of perceived stress. These results differ from the general tendency to believe that both physical and mental flexibility diminish with age [27,28]. However, the results are in line with studies in which workers’ experience has been seen to equip them to better use their coping resources, thanks to the confidence and maturity brought by age and experience [29,30].

As indicated previously, literature agrees that burnout has been used as a concept which operationalizes a significant aspect of perceived stress in teachers, and its negative impact [31], which is expressed in the three components of the syndrome. In this study, perceived stress was a very important variable in burnout. In fact, 31.3% of the variance of burnout was found to be explained by perceived stress. Higher levels of perceived stress were found to be associated with greater Emotional Exhaustion, increased Depersonalization, and lower Personal Accomplishment. 

Emotional Exhaustion was found in female teachers, in line with the studies by Kabadayi [19] and by Hendrix, Acevedo and Hebert [14]. Some authors have explained these differences based on different coping strategies deployed by men and women in the face of stressful experiences [32,33]. These differences may also be due to what Oramas, Almirall and Fernández [31] concluded in their work, indicating that teaching as a profession has a marked gender determinism, which is identified with care and protection, part of the feminine role [34,35,36].

In a similar way to the results about perceived stress, teachers with less experience (less than 10 years) and teachers with more experience (30 years or more) had the lowest scores in Emotional Exhaustion. This is in line with studies into the relationship between burnout and other variables such as experience, for example Taris, Schaufeli, Schreurs and Caljé [37]. However, it contradicts some studies such as Guedes and Gaspar [22], who reported seeing higher levels of Emotional Exhaustion and Depersonalization in older teachers.

The present study did have some limitations. The most important is to do with how some of the variables were assessed. For example, the Burnout Syndrome dimensions were assessed using self-reported accounts. In addition, the method used for data collection (online instruments) made it more challenging for respondents to get timely information or explanations about any doubts or questions they may have had. The sample selection was carried out by sending the survey via email to all teachers with an email address in all Portuguese universities in the North, center and South of the country. This produced a larger sample than necessary to be considered probabilistic. To meet that requirement would have needed 380 respondents for a sampling error of 5% and a 95% confidence level. Our final sample was made up of 520 respondents. Thus, we can infer from the results that there is a high probability of generalization to teachers at Portuguese HEIs, and that this study produced useful results for research and understanding of the subject.

Brito [38] mentions that teaching is a risky condition, which can lead to exhaustion, indicating that there is a need to implement prevention programs to improve participants’ health and safety, concomitantly improving their working conditions [38]. In this regard, our study reinforces a number of implications reported by recent studies [39], which recommend that within a healthy lifestyle, the implementation of programs of regular physical exercise for teachers, accompanied by the development of self-efficacy skills and stress control. In addition, it is suggested that supervising institutions take measures to encourage these practices and reduce the problem of stress. When stressful situations are difficult to control, an appropriate prevention strategy could be improving self-efficacy and stress management programs, for example, by developing prevention/training plans that enhance stress management skills. We therefore underline the importance of carrying out studies of this type [40], which identify the levels of burnout and stress in teachers and the variables that affect them so that actions can be taken to facilitate the elimination of the symptoms of burnout syndrome in this population. This becomes even more important if one considers the fact that work-related stress has high costs on both human and economic levels every year, since it affects people’s physical and psychological health and has a negative impact on organizations, causing absenteeism, low productivity, low performance, low motivation, and discouragement [41]. 

As in previously published systematic reviews [42], our future research will aim to show the great variability existing in the factors studied around teacher burnout in Portugal, in order to extend our research and make more exact comparisons about this construct. Taking into account the limitations described above, our future research will review other studies and include other important variables which we believe should be investigated.

## 5. Conclusions

The great concern in recent times to reduce stress and burnout in workers in all types of organizations make studies like this entirely necessary. In this regard, and in the light of our results, we make the following conclusions according to the specific characteristics of our sample. 

In terms of perceived stress, our results indicate that university teachers who are over 60 exhibit the lowest levels of perceived stress. Another notable result is that teachers with the most (more than 30 years) and least (less than 10 years) experience exhibit the lowest levels of perceived stress. Female teachers exhibit higher levels of perceived stress than male teachers.

In terms of the burnout dimensions, the highest levels of perceived stress were found to be associated with greater Emotional Exhaustion, greater Depersonalization, and lower Personal Accomplishment. Women seemed to exhibit significantly higher levels of Emotional Exhaustion than men. Similarly to perceived stress, teachers with the greatest (30 years or more) and least (less than 10 years) experience had the lowest scores in Emotional Exhaustion.

The conclusion is that perceived stress is a very important variable in burnout. Therefore, coping strategies may be a useful route for stress not to become a true determining factor of burnout.

## Figures and Tables

**Table 1:** Distribution of participants.

**Sex**	***n* (%)**
Men	339 (65.2%)
Women	181 (34.8%)
**Participants’ Age**	***n* (%)**
Under 40	104 (20%)
40 to 49	197 (37.9%)
50 to 59	182 (35%)
Over 60	37 (7.1%)
**Years of Teaching Experience**	***n* (%)**
Less than 10	117 (22.5%)
10 to 19	210 (40.4%)
20 to 29	143 (27.5%)
30 or more	50 (9.6%)
**Knowledge Areas**	***n* (%)**
Social Sciences	158 (30.4%)
Health Sciences	139 (26.7%)
Technological Sciences	124 (23.8%)
Arts and Humanities	76 (14.6%)
Others	23 (4.4%)
**University Type**	***n* (%)**
Public	396 (76.2%)
Private	124 (23.8%)
**Geographical Area**	***n* (%)**
North	216 (41.5%)
Center	159 (30.6%)
South	145 (27.9%)

**Table 2:** Descriptive statistics in Perceived Stress depending on sex, age, teaching experience and knowledge areas taught.

	Perceived Stress	
**Sex**	***M***	***SD***
(1) Women	1.93	0.70
(2) Men	1.74	0.73
**Participants’ Age**	***M***	***SD***
(1) Under 40	1.92	0.71
(2) 40 to 49	1.93	0.71
(3) 50 to 59	1.82	0.71
(4) Over 60	1.62	0.72
**Teaching Experience**	***M***	***SD***
(1) Less than 10 years	1.77	0.64
(2) 10 to 19 years	1.95	0.74
(3) 20 to 29 years	1.89	0.71
(4) 30 years or more	1.68	0.74
**Knowledge Areas Taught**	***M***	***SD***
(1) Social Sciences	1.88	0.73
(2) Health Sciences	1.85	0.67
(3) Technological Sciences	1.85	0.72
(4) Arts and Humanities	1.89	0.73
(5) Others	1.88	0.80

Analysis of Variance (ANOVA) DMS post hoc analysis: Sex (1–2); Participants’ age (1–4, 2–4); Teaching experience (1–2, 2–4).

**Table 3:** Descriptive statistics in the three dimensions of burnout according to perceived stress, sex, age and years of service of the participants.

	Emotional Exhaustion		Depersonalization		Personal Accomplishment	
**Perceived Stress**	***M***	***SD***	***M***	***SD***	***M***	***SD***
(1) Low	1.85	0.91	0.59	0.76	5.12	0.80
(2) Medium	2.84	0.95	0.98	0.80	4.65	0.83
(3) High	4.08	1.01	1.59	1.14	4.34	0.84
**Sex**	***M***	***SD***	***M***	***SD***	***M***	***SD***
(1) Women	3.07	1.33	1.01	0.99	4.67	0.88
(2) Men	2.77	1.32	1.18	1.01	4.73	0.90
**Participants’ age**	***M***	***SD***	***M***	***SD***	***M***	***SD***
(1) Under 40	2.88	1.36	1.28	1.24	4.79	0.84
(2) 40 to 49	3.08	1.34	1.03	0.90	4.66	0.85
(3) 50 to 59	2.99	1.31	1.01	1.02	4.67	0.89
(4) Over 60	2.49	1.26	0.94	0.79	4.65	1.13
**Teaching experience**	***M***	***SD***	***M***	***SD***	***M***	***SD***
(1) Less than 10 years	2.53	1.26	1.05	1.16	4.82	0.86
(2) 10 to 19 years	3.14	1.33	1.10	0.99	4.68	0.83
(3) 20 to 29 years	3.18	1.34	1.06	0.90	4.63	0.93
(4) 30 years or more	2.66	1.25	0.98	1.11	4.61	1.01

ANOVA DMS post hoc analysis: EMOTIONAL EXHAUSTION: Perceived Stress (1–2, 1–3, 2–3); Sex (1–2); Teaching experience (1–2, 1–3, 2–4, 3–4). DEPERSONALIZATION: Perceived Stress (1–2, 1–3, 2–3). PERSONAL ACCOMPLISHMENT: Perceived Stress (1–2, 1–3, 2–3).
